# Antidepressant effects of novel positive allosteric modulators of Trk-receptor mediated signaling – a potential therapeutic concept?

**DOI:** 10.1007/s00213-023-06410-x

**Published:** 2023-07-03

**Authors:** Nather Madjid, Veronica Lidell, Gunnar Nordvall, Maria Lindskog, Sven-Ove Ögren, Pontus Forsell, Johan Sandin

**Affiliations:** 1 AlzeCure Pharma AB, Hälsovägen 7, 141 57, Huddinge, Sweden; 2 Division of Neuroscience, Care and Society, Department of Neurogeriatrics, Karolinska Institutet, Stockholm, Sweden; 3 Department of Neuroscience, Karolinska Institutet, Stockholm, Sweden

**Keywords:** MDD, BDNF, TrkB, Positive allosteric modulator, Ketamine, Fluoxetine, Mice

## Abstract

**Background:**

Major depressive disorder (MDD) is defined as a complex mental disorder which is characterized by a pervasive low mood and aversion to activity. Several types of neurotransmitter systems e.g. serotonergic, glutamatergic and noradrenergic systems have been suggested to play an important role in the origination of depression, but neurotrophins such as brain derived neurotrophic factor (BDNF) have also been implicated in the disease process.

**Objectives:**

The purpose of this study was to examine the effects of a newly developed class of molecules, characterized as positive allosteric modulators of neurotrophin/Trk receptor mediated signaling (Trk-PAM), on neurotransmitter release and depression-like behavior in vivo.

**Methods:**

The effect of and possible interaction of neurotrophin/Trk signaling pathways with serotonergic and glutamatergic systems in the modulation of depression-related responses was studied using newly developed Trk-PAM compounds (ACD855, ACD856 and AC26845), as well as ketamine and fluoxetine in the forced swim test (FST) in rodents. Moreover, in vivo microdialysis in freely moving rats was used to assess changes in neurotransmitter levels in the rat.

**Results:**

The results from the study show that several different compounds, which all potentiate Trk-receptor mediated signaling, display antidepressant-like activity in the FST. Moreover, the data also indicate that the effects of both fluoxetine and ketamine in the FST, both used in clinical practice, are mediated via BDNF/TrkB signaling, which could have implications for novel therapies in MDD.

**Conclusions:**

Trk-PAMs could provide an interesting avenue for the development of novel therapeutics in this area.

## Introduction

Clinical depression or Major depressive disorder, MDD, is one of several psychiatric disorders affecting mood, along with mania, hypomania, and bipolar disorder. Depression is defined as a common complex mental illness which is characterized by a pervasive low and sustained depressive mood, diminished interest and suicidal ideation. Depression is a life-threatening psychiatric disorder and a major public health concern worldwide with an incidence of 5% and a lifetime prevalence of 15–20% (Kessler et al. 2005). Moreover, depression is associated with disability, decreased quality of life, increased health-related costs and is considered a major risk factor for many diseases, including cardiovascular, metabolic and neuropsychiatric disorders (Cryan and Holmes 2005; Thase 2006). Although the currently available antidepressants provide a measurable degree of therapeutic relief, approximately 50% of individuals diagnosed with MDD do not respond adequately to first-line treatment (treatment-resistant MDD patients) with conventional antidepressants (Trivedi et al. 2006; Fava et al. 2008). Current pharmaco-therapeutic treatments have limited efficacy and are associated with many deleterious side effects (Dording, et al. 2002; Lam and Kennedy 2004). Moreover, the 3–4-week delay in the onset of therapeutic efficacy is particularly difficult for patients with persistent suicidal ideation. Therefore, there is a pressing medical need to develop rapidly acting antidepressants that are capable of immediately relieving the depressive symptomology, and persisting in their action as an antidepressant, for treatment of resistant patients unable to respond to conventional therapies. A better understanding of the pathophysiology of this disorder alongside with the development of innovative and improved treatments remains crucial. Hence, animal models are essential for advancing research in this field and screening of novel antidepressants is an important practice in modern research due to the limited efficacy and large number of side effects of existing treatments (Kessler et al. 2003; Licinio and Wong 2005).

One prevailing hypothesis in the pathogenesis of depression has been the monoamine hypothesis, which predicts that the underlying pathophysiologic basis of depression is a depletion in the levels of the monoamines (serotonin, noradrenaline, dopamine) in the central nervous system. However, more recent data rather suggest that imbalances in the levels of biogenic amines, such as dopamine (DA) and serotonin (5-HT), are involved in the etiology of psychiatric disorders like schizophrenia and depression (Sánchez et al. 2010; Sghendo and Mifsud 2012; Czéh et al. 2016). These imbalances in dopaminergic and serotonergic systems are, in turn, likely to affect the chemical balance within the entire neurotransmitter system (Qi et al. 2013; 2016). Furthermore, many studies have shown that this hypothesized pathophysiology appears to be supported by the mechanism of action of antidepressants: agents that alter the levels of the monoamines in the brain have all been shown to be effective in the alleviation of depressive symptoms. Accordingly, therapies for treating depression were derived to target the monoaminergic systems, with a majority of them specifically targeting the serotonin system, with increased serotonin concentrations as a common effect. Although several lines of evidence suggest that alterations in the serotonergic system in the central nervous system may underlie the pathophysiology of depression (Mann 1999; Mikael et al. 2018) half of the MDD patients fail to respond to treatment with a selective serotonin reuptake inhibitor (SSRI), the first line of pharmacological treatment. Furthermore, with antidepressants such as SSRI’s there is a lag time of weeks between initiation of treatment and significant antidepressant effect (Gelenberg and Chesen 2000).

Accumulating evidence have also suggested that glutamatergic neurotransmission plays an important role in the neurobiology and treatment of MDD (Berman et al. 2000; Zarate et al. 2006; Mathew et al. 2012). In fact, clinical studies have demonstrated that the non-competitive N-methyl-d-aspartate (NMDA) receptor antagonist ketamine has rapid antidepressant effects in treatment-resistant patients with MDD (Diazgranados et al. 2010). However, due to problematic side-effects including dissociative effects, sedation and nausea, it is used in some countries only as third line treatment.

Among the alternative mechanisms and therapeutics that have generated increased attention are the neurotrophins, brain derived neurotrophic factor (BDNF) and nerve growth factor (NGF). Neurotrophins are a closely related family of proteins in the brain that contributes to the survival, growth, and maintenance of neurons (Cowansage et al. 2010) and participate in a variety of brain functions such as depression, learning and memory (Ahlskog et al. 2011; Lee and Kim 2010). The mammalian neurotrophins include BDNF, nerve growth factor, neurotrophin 3, and neurotrophins 4/5. Undoubtedly, the majority of the literature linking neurotrophins with depression involves the study of BDNF, and there is in fact a well-established body of clinical evidence implicating the involvement of BDNF in the pathobiology of depression (Lee and Kim 2010). Peripheral reductions in mature BDNF in serum and plasma have been noted in persons with depression (Lee et al. 2007; Yoshida et al. 2012) and in cases of suicide (Birkenhager et al. 2012; Kim et al. 2007). Findings from a meta-analysis and systematic review showed significantly lower levels of serum BDNF in antidepressant-free patients with MDD as compared to healthy controls (Molendijk et al. 2014). Moreover, serum levels of BDNF tend to normalize in response to several treatments with antidepressants, such as sertraline, escitalopram, or venlafaxine (Matrisciano et al. 2009), electroconvulsive therapy (Brunoni et al. 2014), and physical activity (Engesser-Cesar et al. 2007).

Interestingly, several lines of evidence have also indicated that BDNF is required for the antidepressant response to ketamine and SSRI’s, such as fluoxetine. Preclinical studies have found that an infusion of a BDNF- neutralizing antibody into the mPFC abolishes ketamine's antidepressant-like effects, also suggesting that BDNF in the mPFC may be an important site of action (Lepack et al. 2014). Mice expressing the human *BDNF* val66met polymorphism (resulting in lowered BDNF secretion; Egan et al. 2003) have been examined following acute ketamine administration and shown to have an attenuated antidepressant response (Liu et al. 2012), suggesting that functional BDNF is required for the rapid antidepressant action of ketamine. Essentially all antidepressants, including ketamine and SSRI’s, also increase the expression and signaling of brain-derived neurotrophic factor (BDNF) through its receptor, the tyrosine kinase receptor B (TrkB) (Autry and Monteggia 2012; Castrén and Antila 2017; Duman and Monteggia 2006). Moreover, BDNF mimics the effects of antidepressants in rodents and inhibiting TrkB signaling have been shown to prevent their behavioral effects (Duman and Monteggia 2006; Saarelainen et al. 2003). The effects of SSRIs and ketamine on BDNF signaling have been considered to be indirect, through inhibition of the serotonin transporter (5HTT) and NMDA receptors, respectively. However, recent data have shown that both ketamine and SSRI’s such as fluoxetine can bind directly to the TrkB-receptor to mediate their antidepressant effects (Casarotto et al. 2021).

Treatment with small molecules that directly enhance neurotrophin signaling is a mechanism that is less likely to suffer from the inherent drawbacks of using neurotrophic proteins per se, including costly and sometimes complicated treatments, a short half-life in plasma, local adverse events at the site of injection, and inability to pass the blood brain barrier [27]. Several attempts to identify and develop small molecule activators of neurotrophin signaling have been made, including peptidometics [28–35], small molecules with agonistic properties such as 7,8-dihydroxyflavone [36], gambogic amide [37], amitriptyline [38], LM22A [39] AIT-082 [40,41], L-783,281 and its analogues [42,43], MT-2 [44], macrocyclic compounds such as NG-011 and NG-012 [45], various natural products [46], and several both natural and synthetic substances containing a steroid backbone [47–49]. However, very few compounds have so far reached the stage of clinical development.

In our attempts to identify small molecules that can affect the signaling of neurotrophins, we have identified a novel class of compounds acting as positive allosteric modulators (PAM’s) of the Trk-receptors, enhancing the signaling of the endogenous ligands such as BDNF and NGF. These compounds have been shown to enhance synaptic plasticity as well as attenuate cognitive dysfunction in a TrkB-receptor dependent manner in several different behavioral animal models, including the passive avoidance model, water maze and object recognition task (Dahlström et al. 2021). The first molecule to be developed in this novel chemical class was ACD855, which was discontinued due to a long half-life in man. The subsequent compound, ACD856, recently completed the phase I clinical trials and AC-0026845 is a discovery compound in research phase. Although the compounds are structurally and pharmacologically very similar in the in vitro assays, the use of multiple Trk-PAM compounds with slightly different physicochemical and pharmacokinetic properties was deemed appropriate to better profile this new class of molecules with respect to their potential antidepressant-like effects.

This study aimed to examine the effect of different PAM’s of Trk-receptors in FST-induced depression-like behavior in rodents. Here, we used several Trk-PAM’s compounds, ACD855, ACD856, AC26845 as well as ANA-12, a TrkB antagonist (Cazorla et al. 2011), as pharmacological tools. We examined the effects of these compounds on depression-like behavior and brain neurotransmitter levels, using in vivo microdialysis. Moreover, the potential interaction between a Trk-PAM compound and ketamine or fluoxetine in the modulation of depressive-like behaviors was also assessed.

## Materials and methods

### Animals

Adult male C57BL/6 J mice (Charles River, Germany) were used in all experiments. The mice were 7–8 weeks of age and weighed 25–30 g at the time of testing. The animals were housed in groups of 4–6 mice in standard Macrolon® cages (A3, 42 × 26x20 cm) in a temperature- and humidity-controlled room (21 ± 1ºC and 60 ± 5% humidity), with a 12-h light/dark cycle (lights on at 6.00 am), with free access to standard lab chow (Ewos R36, Ewos AB, Södertälje, Sweden) and tap water were provided *at libitum*. The animals were allowed to habituate to the maintenance facilities for a period of at least seven days before the experiments. The animals were transferred to the experimental room one hour prior to the experiment for habituation and all animals were handled and tested by a single researcher. All experiments were conducted in experimentally naïve animals during the light phase (between 9 am and 3 pm), with all efforts made to minimize suffering and the number of animals used. The cages were changed once a week. Animal housing and experimental procedures followed the protocols and recommendations of the Swedish animal protection legislation. The experimental procedures were approved by the local Animal Ethics Committees (ID 1640) conformed to the European Council Directive (2010/63/EU).

To study the effect in rats, male Flinders sensitive line (FSL) rats was chosen. This selectively bred rat strain is derived from the Sprague–Dawley strain and associated with distinct behavioral and neurochemical features of major depression, including some which have face validity, e.g. psychomotor retardation and increased rapid eye movement sleep. FSL rats display a genetic vulnerability to environmental stressors and have been successfully used for validation of antidepressant effects (Overstreet et al. 2005). These animals were bred at the Department of Physiology and Pharmacology at Karolinska Institute and were tested at 2–4 months of age. Rats were kept in standard cages (TypeIV Macrolon, Bayer Material Science, Leverkusen, Germany, 26 × 42 × 15 cm) at room temperature and relative humidity (45–55%) under a constant light/dark cycle (lights on at 06.00 h). Water and food pellets (LactaminR36, Stockholm, Sweden) were available ad libitum. FSL rats were randomly assigned to groups given either fluoxetine, ACD855, ACD856 or vehicle.

The microdialysis experiments were conducted at Pronexus AB, Bromma, Sweden (ethical permit N24/14) in awake, freely moving male Sprague Dawley rats (n = 6, 7–8 weeks old from Janvier Labs, Le Genest-Saint-Isle, France). Rats were maintained in a controlled environment (22 ± 1 °C; 45–50% rel. humidity) on a 12 h dark/12 h light (40 Lux) cycle. The rats had free access to standard lab chow (RM1A(P), SDS, Scanbur, Sweden) and tap water during the entire housing period and during the microdialysis experiments. The rats were examined and weighed prior to initiation of the study to assure adequate health and suitability.

### Drugs and drug administration

ACD855, ACD856 and AC-0026845 were supplied by AlzeCure Pharma and, like ANA 12 (Tocris), dissolved in 20% dimethylsulfoxide (DMSO) in 0.1 M phosphate-buffered (PBS). s-Ketamine (Sigma-Aldrich, Stockholm, Sweden) and fluoxetine hydrochloride (Sigma-Aldrich, Stockholm, Sweden) were dissolved in saline. For background data of ACD855 and ACD856, please see Dahlström et al. (2021). AC-0026845 displays an IC50 of 106 nM on TrkB, 196 nM on TrkA and 150 nM on TrkC in the same primary assay as used with ACD855 and ACD856. The doses chosen were based on the known pharmacokinetics of the compounds. All drugs were injected at a volume of 8 ml/kg intraperitoneally (i.p.) or subcutaneously (s.c.) in the scruff of the neck. All drugs were administered either acutely (single dose) or repeated doses (once daily) for 4 or 28 consecutive days with the last injection performed 30–60 min prior to the experiment.

### Forced swim test (FST)

The FST is the most frequently used behavioral test for measuring depressive-like behavior in rodents (Porsolt et al. 1977, 1978). Animals placed in cylinders containing water rapidly become immobile, demonstrated by floating passively or making only movements necessary to remain afloat. Based on an immobility response induced by inescapable exposure to stress, the FST also has strong predictive validity because short-term administration of antidepressant compounds from a variety of pharmacological classes reduces immobility time in the FST. These drugs include tricyclic antidepressants, MAO inhibitors, atypical antidepressants, and SSRIs (Cryan et al. 2005a, b).

Depression-like behavior was assessed in both mice and rats, using a modified version of the FST, as described previously (Kuteeva et al. 2005). This included a two day protocol test, with pre-exposure to water 24 h prior to the test (day one of FST), which seems to be a more accurate and sensitive technique in detecting depression-like behavior in mice than the standard method of a single exposure (one day test) (Kuteeva et al. 2005; Porsolt et al. 1977; Dalvi and Lucki 1999). Animals were individually placed in a vertical glass cylinder (50 cm high, 20 cm in diameter, CMA) filled with tap water up to 35 cm (25 ± 0.5 °C). Two swimming sessions were conducted: a 10 min pre-test (day one) followed 24 h later (day two) by a 6 min test session. The total duration of immobility as well as latency to the first immobility were recorded during the 6 min test. After each swimming session, the mice were gently removed and placed in the home cage together with dry napkins. Immobility was defined as floating passively in an upright position in the water, with only small movements necessary to keep the head above the water surface. The floating time was considered as an index of depression-like behavior. The glass cylinder was cleaned thoroughly between each animal.

### In vivo microdialysis

The microdialysis experiments were conducted at Pronexus AB, Bromma, Sweden (ethical permit N24/14) in awake, freely moving male Sprague Dawley rats as previously described (Kehr et al. 1998; Yoshitake et al. 2004).

Briefly, rats were anesthetized with isoflurane, placed in a stereotaxic frame and a guide cannula (Eicom Corp., Kyoto, Japan) was implanted into the hippocampus at the following coordinates: AP—5.2 mm, L + 5.0 mm, V—2.8 mm, providing the final -6.8 mm for the tip of the microdialysis probe. The guide cannula was fixed firmly to the skull surface using dental cement (Dentalon Plus, Heraeus Instruments, Hanau, Germany) and the scalp incision was closed with sutures after which the animals were allowed to recover for a week.

On the day of the microdialysis experiment, the rat in its home cage was placed into the frame of a Rotating animal cage system, RACS (Microbiotech/se, Årsta, Sweden). The RACS allows a swivel-free connection of the microdialysis tubing to each respective syringe pump and to the fraction collector and at the same time, brief recording of locomotor activation. A microdialysis probe (Eicom A-I: 0.22 mm O.D., 4 mm membrane length with cut-off 50 kDa) was inserted into the guide cannula of the awake rat. The probe was connected with fluorinated ethylene propylene (FEP) tubing (0.1 mm I.D.) to the balancing arm of the RACS system. The probe was perfused at a constant flow-rate of 1 μl/min with artificial cerebrospinal solution (aCSF: 148 mM NaCl, 4 mM KCl, 0.8 mM MgCl2, 1.4 CaCl2, 1.2 mM Na2HPO4, 0.3 mM NaH2PO4, pH 7.2). Each rat was allowed to habituate to the new environment for 120 to 150 min. Following this stabilization period, the microdialysis samples were collected in 30-min intervals. The first 3 samples were collected for determination of basal extracellular levels of neurotransmitters. Thereafter, ACD856 or vehicle formulation was administered s.c. and the samples were collected for an additional 24 h. After finalizing the experiment, the animals were sacrificed by an overdose of isoflurane and dislocation of the neck. The brains were rapidly removed, flash-frozen on dry ice and stored at -80 °C for additional analysis of tissue biomarkers and histological verification of the microdialysis probe placement.

Concentrations of acetylcholine (ACh) and glutamate (Glu) in the microdialysis samples were measured using ultra-high performance liquid chromatography tandem mass spectrometry (UHPLC-MS/MS), whereas the levels of the monoamines dopamine (DA), noradrenaline (NA), and 5-serotonin (5-HT) in the microdialysates were measured using ion-exchange narrow bore column liquid chromatography with electrochemical detection as described elsewhere (Kehr et al. 1998, 2007; Yoshitake et al. 2004).

### Data analysis

The results were analyzed using a one-way analysis of variance (ANOVA) with treatment as between group factors. If significant, Tukey’s multiple comparison test was performed to assess statistical difference between the groups. Unpaired student t-test was used in experiments with only two groups. The level of significance was set at 0.05. The study was designed as a between subjects (independent groups) experiment (i.e. each animal was used only once). Where relevant, a two-way ANOVA was used to assess potential synergistic effects of combinatorial treatments. All statistics and corresponding graphs were made using Graph Pad Prism. Data are reported as mean values ± standard error of the mean (SEM).

## Results

### Single and repeated administration of ACD855

**Fig. 1:**
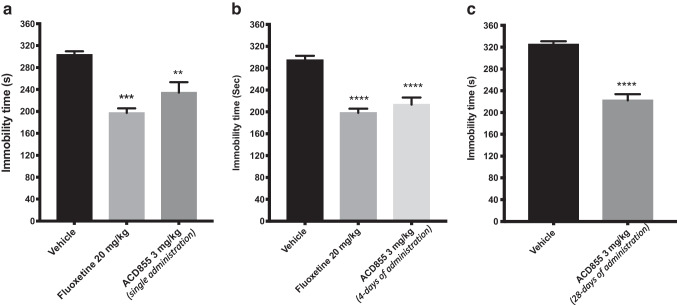
Antidepressant-like effects in the forced swim test after ACD855 administration. The forced swim test was conducted using a 10 min pre-test (day 1) followed 24 h later (day 2) by a 6 min test session where the immobility was recorded. Mice were injected either with a *single dose* of ACD855 (3 mg/kg, s.c.; panel a) on day 2 prior to behavioral test or *repeated administration* (once daily; 3 mg/kg/day, s.c.) for 4 or 28 consecutive days (panel b and c respectively) before behavioral testing, and were compared with mice receiving vehicle or mice treated with a *single dose* of 20 mg/kg fluoxetine on day 2 prior to behavioral test. Both fluoxetine and ACD855 (p < 0.01; p < 0.001; p < 0.0001) significantly reduced the immobility time in the forced swim test compared with vehicle treated mice. The bars represent the immobility time (seconds), mean ± SEM (n = 6–8 mice per group). The statistical analysis was performed using one-way ANOVA followed by Tukey's test (panel A and B), or unpaired t-test (C). **p < 0.01; ***p < 0.001; ****p < 0.0001 vs control group

The effect of single, and repeated injections of the Trk-PAM ACD855 on depression-like behaviors in mice is shown in Fig. 1. An overall statistically significant effect of a single treatment was observed with respect to immobility time (considered as an index of depression) using one-way ANOVA (*F(*2,15) = 13.93; p < 0.001). Post hoc analysis revealed that mice receiving a single injection of 3 mg/kg of ACD855 displayed a significantly shorter immobility time compared with control mice (Fig. 1a, p < 0.01). The average immobility time for control mice was 304 s compared with an average immobility time of 234 s for the ACD855 treated mice. Similar results were shown for mice treated with a single dose of the selective serotonin reuptake inhibitor (SSRI), fluoxetine at 20 mg/kg, with a significantly lower immobility time (197 s) than the control mice (Fig. 1a, p < 0.001).

The effect of repeated administration of ACD855 for 4 days or 28 days showed an overall statistically significant effect of treatment regarding immobility time using one-way ANOVA (*F* (3, 23) = 17.90; p < 0.0001 and P < 0.0001, unpaired t-test). Post hoc analysis revealed that mice receiving daily injections for 4 consecutive days with 3 mg/kg/day of ACD855, or 3 mg/kg/day for 28 days displayed a significantly shorter immobility time compared with control mice (Fig. 1b; p < 0.0001, after 3 mg/kg for 4 days respectively; and p < 0.0001 after 3 mg/kg/day for 28 days, Fig. 1c).

### Single and repeated administration of ACD856

**Fig. 2:**
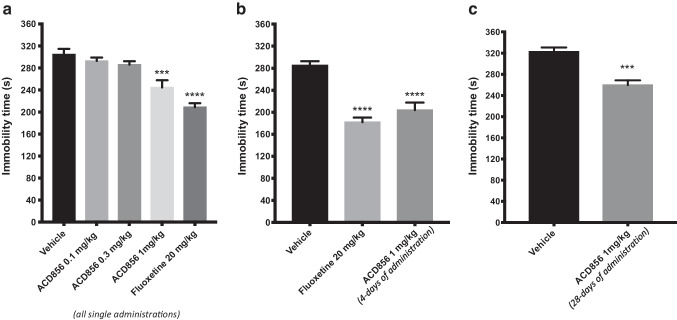
Antidepressant-like effects in the forced swim test after ACD856 administration. The forced swim test was conducted using a 10 min pre-test (day 1) followed 24 h later (day 2) by a 6 min test session where the immobility was recorded. Mice were injected either with a *single dose* of ACD856 (0.1, 0.3 and 1 mg/kg, s.c.; panel a) on day 2 prior to behavioral test or *repeated administration* (once daily; 1 mg/kg/day, s.c.) for 4 and 28 days (panel b and c respectively) before behavioral testing, and were compared with vehicle treated mice or mice treated with a *single dose* of 20 mg/kg fluoxetine on day 2 prior to behavioral test. Both fluoxetine (p < 0.0001) and ACD856 (p < 0.001; 0.0001) significantly reduced the immobility time in the forced swim test compared with vehicle treated mice. The bars represent the immobility time (seconds), mean ± SEM (n = 6–8 mice per group). The statistical analysis was performed using one-way ANOVA followed by Tukey's test (panel A and B), or unpaired t-test (panel C). ***p < 0.001; ****p < 0.0001 vs control group

The effect of single and repeated injections of the Trk-PAM ACD856 on depression-like behaviors in mice is shown in (Fig. 2). An overall statistically significant effect of single treatment was observed regarding immobility time using one-way ANOVA (*F(*4, 25) = 19.30; p < 0.0001). Post hoc analysis revealed that mice receiving a single injection of 1 mg/kg of ACD856 displayed a significantly shorter immobility time compared with control mice (Fig. 2a, p < 0.001). The average immobility time for control mice was 305 s compared with an average immobility time of 245 s for the ACD856 (1 mg/kg) treated mice. Similar results were also seen in mice treated with higher doses of ACD856 (10 and 50 mg/kg; p < 0.001; data not shown). The selective serotonin reuptake inhibitor (SSRI), fluoxetine at 20 mg/kg, i.p. induced a highly significant decreased immobility time (209 s) compared to the control mice (Fig. 2a, p < 0.0001).

The effect of repeated administration of ACD856 (1 mg/kg) for 4 days or 28 days injection was also evaluated in the mouse FST. An overall statistically significant effect of treatment was observed regarding immobility time using one-way ANOVA (*F* (2, 20) = 25.93; p < 0.0001 and P < 0.0001, unpaired t-test) after 4 or 28 days of injections respectively. Post hoc analysis revealed that mice receiving daily injections of ACD856 for 4 or 28 consecutive days at a dose of 1 mg/kg/day displayed a significantly shorter immobility time compared with control mice (Fig. 2b; p < 0.0001; after 1 mg/kg for 4 days) and (Fig. 2c; p < 0.001 after 1 mg/kg/day for 28 days,).

### Effect of repeated (4 days) injections of ACD855 and ACD856 in Flinders sensitive line (FSL) rats, a rat model of depression

**Fig. 3:**
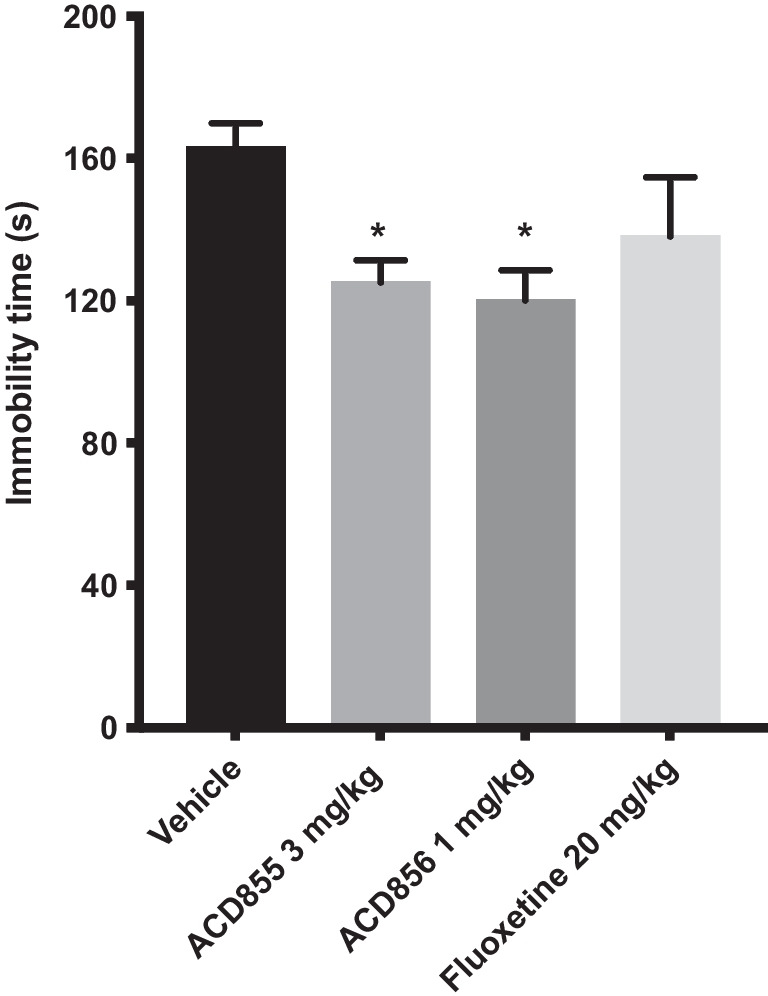
Antidepressant-like effects in the forced swim test after administration of ACD855, ACD856 and fluoxetine. The forced swim test was conducted using a 10 min pre-test (day 1) followed 24 h later (day 2) by a 6 min test session where the immobility was recorded. FSL rats were *repeatedly* (4 days) administered a dose of ACD856 (1 mg/kg/day, s.c.), ACD855 (3 mg/kg/day, s.c.) before behavioral testing, or a *single dose* of fluoxetine (20 mg/kg, i.p.) on day 2 prior to behavioral test and were compared with vehicle treated animals. ACD855 and ACD856 significantly reduced (p < 0.05) the immobility time in the forced swim test compared with control rats. The bars represent the immobility time (seconds), mean ± SEM (n = 7–8 rats per group). The statistical analysis was performed using one-way ANOVA followed by Tukey's test. *p < 0.05 vs control group

FSL rats were randomly assigned to groups given either a single injection of fluoxetine (20 mg/kg, i.p.), a repeated once daily injection for 4 days of ACD855 (3 mg/kg, s.c.) or ACD856 (1 mg/kg, s.c.) or vehicle. An overall statistically significant effect of treatment was observed regarding immobility time using one-way ANOVA (*F* (3, 27) = 3.91; p = 0.0194). Post hoc analysis revealed that rats receiving daily injections of ACD855 for 4 consecutive days at a dose of 3 mg/kg/day and of ACD856 at a dose of 1 mg/kg/day displayed a significantly shorter immobility time compared with control animals (p < 0.05; Fig. 3). A single injection of fluoxetine (20 mg/kg, i.p.) administered 30 min before test failed to induce any significant (p = 0.312, n.s.) effect on immobility in the forced-swim test in FSL rats (Fig. 3).

### Single administration of AC-0026845 alone or in combination with a selective TrKB receptor antagonist ANA-12

**Fig. 4:**
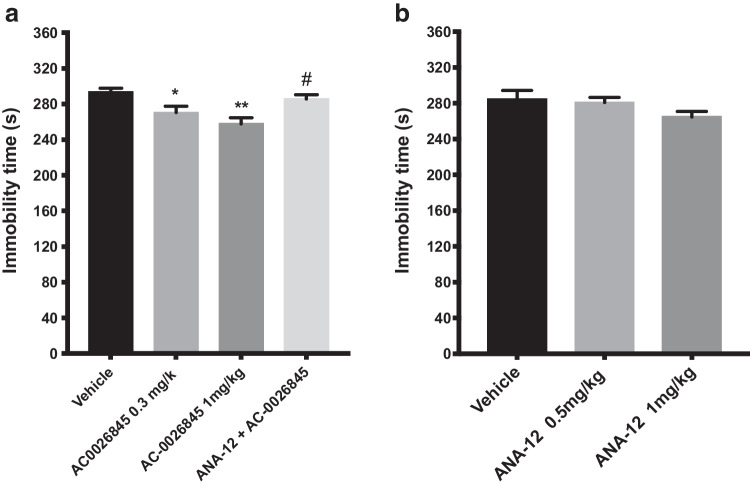
Antidepressant-like effects of AC-0026845 and ANA-12 in the forced swim test. The forced swim test was conducted using a 10 min pre-test (day 1) followed 24 h later (day 2) by a 6 min test session where the immobility was recorded. Mice were injected day 2 prior to behavioral test either with a *single dose* of AC-0026845 (0.3 and 1 mg/kg, s.c.) or AC-0026845 (1 mg/kg, s.c.) and ANA-12 (0.5 mg/kg, s.c., panel a) or with different doses of ANA-12 (0.5 and 1 mg/kg, s.c., panel b) and were compared with control mice or mice treated with a *single dose* of 1 mg/kg of AC-0026845. AC-0026845 induced a significant decrease in immobility time at 0.3 and 1 mg/kg (p < 0.05; 0.01, respectively) compared with control mice. Administration of ANA-12 (0.5 mg/kg) 30 min prior to AC-0026845 (1 mg/kg) completely blocked its antidepressant-like activity (P < 0.05). ANA-12 at the above mentioned doses, failed to induce any significant decrease in immobility time (p = 0.139, ns). The bars represent the immobility time (seconds), mean ± SEM (n = 8 mice per group). The statistical analysis was performed using a two-way ANOVA (Fig. 4a) and one-way ANOVA (Fig. 4b) respectively, followed by Tukey's test. *P < 0.05 and **p < 0.01vs control; #p < 0.05 vs AC-0026845-treated mice

The effect of a single injection of the Trk-PAM AC-0026845 (0.3 and 1 mg/kg, s.c.) alone or in combination with a low dose of ANA-12 (0.5 mg/kg, s.c.) on depression-like behaviors in mice is shown in Fig. 4a. An overall statistically significant effect of a single administration was observed regarding immobility time using two-way ANOVA (*F(*3, 28) = 7.135; p < 0.01). Post hoc analysis revealed that mice receiving a single injection of AC-0026845 (1 mg/kg) displayed a significantly shorter immobility time compared with control mice (p < 0.01; Fig. 4). Mice treated with acute single dose of ANA-12, 30 min prior to AC-0026845 (1 mg/kg) significantly blocked the antidepressant-like effect of AC-0026845 (p < 0.05; Fig. 4a). The two-way ANOVA also revealed a significant interaction of the two treatments (*F(*1, 28) = 15.08; p < 0.01).

Acute single injection of different doses of ANA-12 (0.5 and 1 mg/kg) failed to induce any significant effect on immobility time (p = 0.139, ns; Fig. 4b).

### Effect of a single administration of different doses of fluoxetine alone or in combination with AC-0026845

**Fig. 5:**
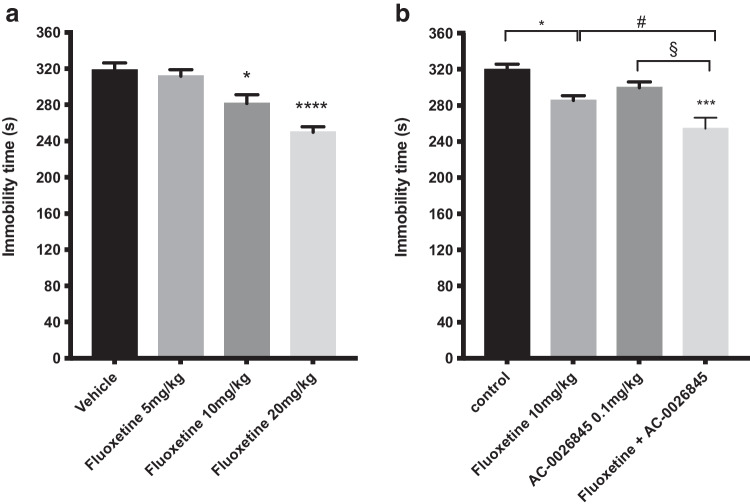
Antidepressant-like effects of different doses of fluoxetine in the forced swim test. The forced swim test was conducted using a 10 min pre-test (day 1) followed 24 h later (day 2) by a 6 min test session where the immobility was recorded. Mice were injected day 2 prior to behavioral test either with acute *single doses* of fluoxetine (5, 10 and 20 mg/kg, i.p.) or administered both fluoxetine (10 mg/kg, i.p.) and AC-0026845 (0.1 mg/kg, s.c.) and were compared with vehicle treated mice or mice given a single dose of fluoxetine (10 mg/kg) or AC-0026845 (0.1 mg/kg). Fluoxetine at doses 5, 10 and 20 mg/kg induced a significant decrease in immobility time at 10 and 20 mg/kg (Fig. 5a, p < 0.05; p < 0.0001, respectively) compared with control mice. Administration of a subthreshold dose of AC-0026845 (0.1 mg/kg) in combination with fluoxetine (10 mg/kg) induced a significant decrease in immobility time as compared to control, AC-0026845 (0.1 mg/kg) or fluoxetine (10 mg/kg) alone (Fig. 5b, p < 0.01, p < 0.05; p < 0.05, respectively). The bars represent the immobility time (seconds), mean ± SEM (n = 6 mice per group). The statistical analysis was performed using one-way ANOVA (Fig. 5a) and two-way ANOVA (Fig. 5b) respectively, followed by Tukey's test. *p < 0.05; ****p < 0.001vs control; #p < 0.05 vs fluoxetine treated mice; §p < 0.05vs AC-0026845 treated mice

The effect of an single injection of the SSRI fluoxetine (5, 10 and 20 mg/kg, i.p.) alone or in combination with a subthreshold dose of AC-0026845 (0.1 mg/kg, s.c.) on depression-like behaviors in mice is shown in Fig. 5. An overall statistically significant effect of acute treatment was observed regarding immobility time using one-way ANOVA (*F(*3, 20) = 15.45; p < 0.0001). Post hoc analysis revealed that mice receiving a single injection of fluoxetine displayed a significantly shorter immobility time compared with control mice (p < 0.05; 0.000, after 10 and 20 mg/kg respectively; Fig. 5a). The combined effect of fluoxetine (10 mg/kg) and AC-0026845 (0.1 mg/kg) on depression-like behaviors in mice is shown in Fig. 5b. An overall statistically significant effect of acute treatment was observed regarding immobility time using two-way ANOVA (*F(*3, 26) = 11.24; p < 0.0001). Post hoc analysis revealed that mice receiving a single injection of fluoxetine (10 mg/kg) displayed a significantly shorter immobility time compared with control mice (p < 0.05; Fig. 5b). Interestingly, combined administration of fluoxetine (10 mg/kg) and a sub-threshold dose of AC-0026845 (0.1 mg/kg) induced a marked decrease in immobility time compared with control mice, fluoxetine and AC-0026845 (p < 0.001 vs control; p < 0.05 vs fluoxetine and p < 0.05 vs AC-0026845, respectively). The two-way ANOVA did not however reveal a significant interaction (*F(*1, 26) = 1.6; ns).

### Effect of a single administration of different doses of ketamine alone or in combination with AC-0026845

**Fig. 6:**
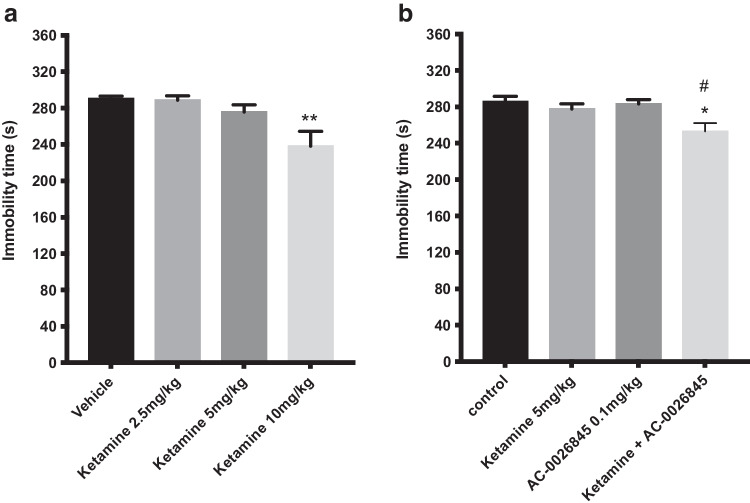
Antidepressant-like effects of different doses of Ketamine in the forced swim test. The forced swim test was conducted using a 10 min pre-test (day 1) followed 24 h later (day 2) by a 6 min test session where the immobility was recorded. Mice were injected day 2 prior to behavioral test either with acute *single doses* of ketamine (2.5, 5, and 10 mg/kg, i.p.) or administered both a sub-threshold dose of ketamine (5 mg/kg, i.p.) and AC-0026845 (0.1 mg/kg, s.c.) and were compared with vehicle treated mice and mice treated with a single dose of ketamine (5 mg/kg) or AC-0026845 (0.1 mg/kg). Ketamine at doses 2.5, 5, and 10 mg/kg induced a significant decrease in immobility time at 10 mg/kg (Fig. 6a, p < 0.01) compared with control mice. Administration of AC-0026845 (0.1 mg/kg) in combination with ketamine (5 mg/kg) induced a significant decrease in immobility time as compared to control and AC-0026845 treated mice (Fig. 6b, P < 0.05; p < 0.05, respectively). The bars represent the immobility time (seconds), mean ± SEM (n = 7–8 mice per group). The statistical analysis was performed using one-way ANOVA (Fig. 6a) and two-way ANOVA (Fig. 6b) respectively, followed by Tukey's test. *p < 0.05 vs control; #p < 0.05 vs AC-0026845 treated mice

The effect of an acute injection of the NMDA receptor antagonist ketamine (2.5, 5 and 10 mg/kg, i.p.) and its combination with AC-0026845 (0.1 mg/kg, s.c.) on depression-like behaviors in mice is shown in (Fig. 6). An overall statistically significant effect of acute treatment was observed regarding immobility time using one-way ANOVA (*F(*3, 26) = 5.98; p < 0.01). Post hoc analysis revealed that mice receiving a single injection of ketamine displayed a significantly shorter immobility time compared with control mice (p < 0.01, after 10 mg/kg; Fig. 6a). The combined effect of sub-threshold dose of ketamine (5 mg/kg) and AC-0026845 (0.1 mg/kg) on depression-like behaviors in mice is shown in Fig. 6b. An overall statistically significant effect of acute treatment was observed regarding immobility time using two-way ANOVA (*F(*3, 28) = 5.29; p < 0.01). Post hoc analysis revealed that neither mice receiving a single injection of ketamine (5 mg/kg) alone nor AC-0026845 (0.1 mg/kg) induced any significant effect on immobility time (p = 0.807; 0.993, n.s., respectively). Interestingly, combined administration of non-effective dose of ketamine (5 mg/kg) and AC-0026845 (0.1 mg/kg) induced a significantly decrease in immobility time compared with control and AC-0026845 treated mice (p < 0.05 vs control, p < 0.05 vs AC-0026845 treated mice Fig. 6b). The two-way ANOVA did not however reveal a significant interaction (*F(*1, 28) = 2.8; ns).

### Effect of a single administration of fluoxetine or ketamine alone or in combination with ANA-12

**Fig. 7:**
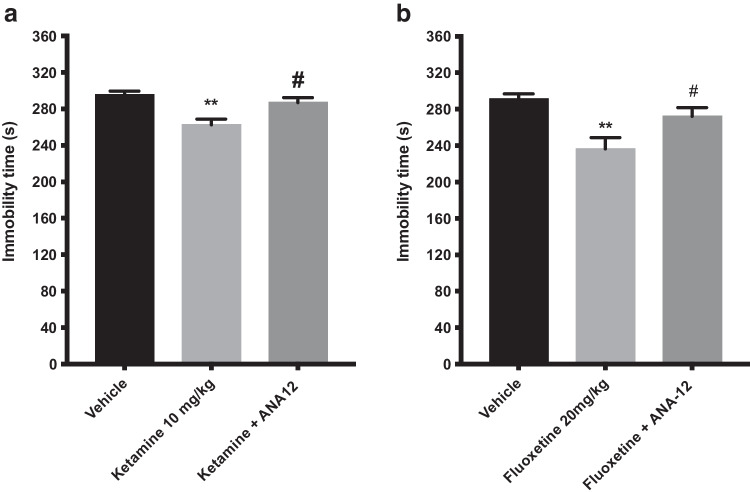
Effect of ANA-12 on Antidepressant-like activity of Ketamine and fluoxetine in the forced swim test. The forced swim test was conducted using a 10 min pre-test (day 1) followed 24 h later (day 2) by a 6 min test session where the immobility was recorded. Mice were injected day 2 prior to behavioral test with a *single dose* of ketamine (10 mg/kg, i.p.) or fluoxetine (20 mg/kg, i.p.), or in combination with ANA-12 (0.5 mg/kg, s.c.) and were compared with vehicle treated mice or mice treated with a single dose of ketamine (10 mg/kg) or fluoxetine (20 mg/kg) alone. Ketamine induced a significant decrease in immobility time at 10 mg/kg (Fig. 7a, p < 0.01) compared with control mice. Administration of ANA-12 (0.5 mg/kg) 30 min prior to ketamine (10 mg/kg) completely blocked its antidepressant-like activity (Fig. 7a, P < 0.05). Fluoxetine (20 mg/kg) induced a significant decrease in immobility time as compared to control mice (Fig. 7b, p < 0.01). Similarly, administration of ANA-12 (0.5 mg/kg) 30 min prior to fluoxetine, completely blocked its antidepressant like activity (Fig. 7b, p < 0.05). The bars represent the immobility time (seconds), mean ± SEM (n = 7–8 mice per group). The statistical analysis was performed using one-way ANOVA followed by Tukey's test. **p < 0.01vs control; #p < 0.05 vs ketamine- or fluoxetine-treated mice

The effect of acute injection of ketamine (10 mg/kg, i.p.) or fluoxetine (20 mg/kg, i.p.) and their co-administration with ANA-12 (0.5 mg/kg, s.c.) on depression-like behaviors in mice is shown in Fig. 7. An overall statistically significant effect of acute treatment of ANA-12 and ketamine was observed regarding immobility time using one-way ANOVA (*F(*2, 19) = 9.222; p < 0.01). Post hoc analysis revealed that mice receiving a single injection of ketamine (10 mg/kg) displayed a significantly shorter immobility time compared with control mice (p < 0.01; Fig. 7a). Mice treated with an acute single dose of ANA-12, 30 min prior to ketamine significantly blocked the antidepressant-like effect of ketamine (10 mg/kg) (p < 0.05; Fig. 7a). An acute injection of ANA-12 (0.5 mg/kg) 30 min prior to fluoxetine (20 mg/kg) on depression-like behaviors in mice is shown in (Fig. 7b). An overall statistically significant effect of acute treatment was observed regarding immobility time using one-way ANOVA (*F (*2, 21) = 8.324; p < 0.01). Post hoc analysis revealed that mice receiving a single injection of fluoxetine (20 mg/kg) displayed a significantly shorter immobility time compared with control mice (p < 0.01; Fig. 7b). Mice treated with ANA-12, 30 min before fluoxetine significantly blocked the antidepressant-like effect of fluoxetine (10 mg/kg) (p < 0.05; Fig. 7B).

### Effect of a single administration of ACD856 on neurotransmitter release as measured by in vivo microdialysis in the rat hippocampus

**Fig. 8:**
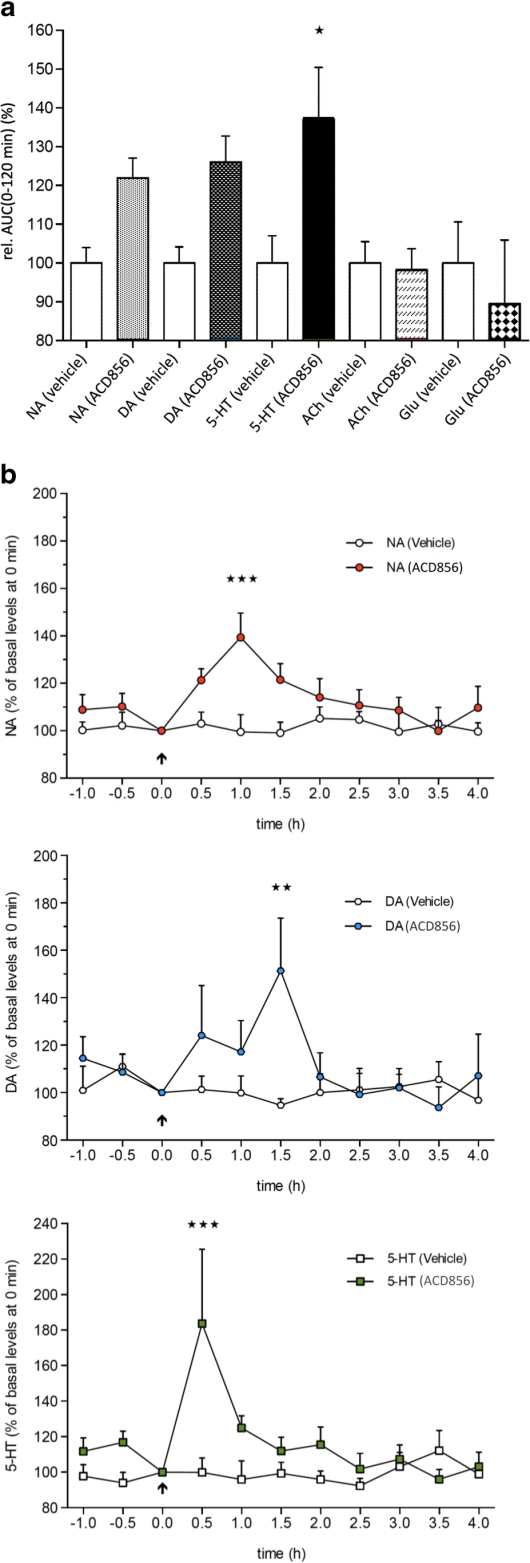
Effects of ACD856 on neurotransmitter release in the hippocampus. Rats were injected with a *single dose* of ACD856 (10 mg/kg) s.c. and serotonin (5-HT), noradrenaline (NA), dopamine (DA), acetylcholine (ACh) and glutamate (Glu) release were continuously measured by microdialysis. There was a significant increase in the amount of serotonin as measured by a) AUC(0-120 min) values, while a more detailed analysis in b) showed a rapid and transient effect. Noradrenaline and dopamine did not reach significance in AUC (a) but showed significant increases at the 60-90 min timepoint (b). No significant effects of ACD856 on the extracellular levels of acetylcholine or glutamate were seen (data not shown). The statistical analysis was performed using a one-way (Fig. 8a) and two-way repeated measures ANOVA (Fig. 8b) respectively, followed by Bonferroni’s multiple comparison test.

The effects of acute administration of ACD856 on extracellular concentrations of NA, DA, 5-HT, Glu and ACh as measured in the microdialysates are shown in Fig. 8a and 8b. The results show a clear trend of ACD856 to increase relative AUC(0-120 min) values for the different monoamines, with 5-HT levels being significantly (P < 0.05) higher than the vehicle group during the first 2 h post-treatment. The effect on 5-HT release was rapid as the levels increased to 184% (P < 0.001) already in the first 30-min samples. The maximum increase (139%) in the NA levels was observed at 60 min, whereas the increase in DA levels was somewhat slower, the peak effect of 151% occurred at 90 min. The effects of ACD856 were transient and the levels of all three monoamines returned to the basal levels 2 h post-dose. No significant effect of ACD856 on the extracellular levels of acetylcholine or glutamate was seen.

## Discussion

The primary symptom of clinical depression is changes in mood, which makes it difficult to develop animal models of clinical depression (Fazer and Morilak 2005). However, various tests of different depression-like behaviors based on exposure to stressful stimuli have been developed, including the learned helplessness model (Seligman et al. 1980), the FST (Porsolt et al. 1977) and the tail suspension test (Steru et al. 1985) to be used with different animal models of depression, e.g. rodents subjected to chronic mild stress (Willner 1997) or FSL rats. The forced swim test is the most widely used test of behavioral despair, i.e. the despair elicited by the exposure to animals to an inescapable situation, and it is based on assumption that rodents will try to escape an aversive stimulus. During the test, animals are under stress from which they cannot escape. After an initial period of struggling, they would become immobile, resembling a state of despair and mental depression. This test is easy, reliable across different laboratories and it has been shown to be sensitive to various factors which are known to influence the development of depression in humans, including genetic predisposition, changes in food intake, alterations in sleep, as well as previous exposure to stress (Cryan et al. 2005a, b). Moreover, FST has a high degree of predictive validity, since it is sensitive to all types of antidepressant treatments, including tricyclic antidepressant (TCA), monoamine oxidase inhibitors (MAOI), selective serotonin reuptake inhibitors (SSRIs), nor-epinephrin reuptake inhibitors (NRIs), NMDA antagonists and electroconvulsive therapy (Borsini and Meli 1988; Berman et al. 2000; Zarata et al. 2006; Petryshen et al. 2016; Khakpai et al. 2019). It has therefore been suggested that there is a functional relationship between this behavioral model and some behaviors elicited by clinical depression (Lucki et al. 2001). However, it is also important to consider the limitations of only using one behavioral model, the FST, in the assessment of depression-like behaviors (Reardon et al. 2019). Complementing behavioral models, such as the sucrose preference test, could add further valuable information on the effects of these compounds in future studies.

An interesting finding of the present study is the decreased time of immobility in the Porsolt forced swim test both in mice and in Flinders sensitive line of rats (FSL) which display a genetic vulnerability to environmental stressors and have been successfully used for validation of antidepressant effects (Overstreet et al. 2005). Interestingly, acute and chronic administration of the positive allosteric modulators (PAMs) of Trk-receptors, ACD855, ACD856 and AC-0026845 displayed reduced immobility time as compared to control group. Given the structural and pharmacological similarities of the compounds, this effect is likely mediated by activation of TrkB receptors, since the anti-depressant effect of AC-0026845 was blocked by pre-administration of the selective TrkB receptor antagonist, ANA-12. In this context, it is interesting to note that we have previously shown that the pro-cognitive effects seen with ACD855 and ACD856 was also blocked by ANA-12 (Dahlström et al. 2021), suggesting that TrkB is a key mediator of both of these in vivo effects for this class of compounds. In our experiments, we did not observe any antidepressant effect of ANA-12 itself, as some other studies have reported previously (Cazorla et al. 2011; Zhang et al. 2014). Based on local microinjection studies, it has been suggested that the site of action for the antidepressant effects of agonists and antagonists differ (Zhang et al. 2014). While stimulation of TrkB in the hippocampus and in the prefrontal cortex conferred antidepressant effects, so did blockade of TrkB in the nucleus accumbens. The discrepancy between these studies and our experiments could be related to the doses used and the subsequent exposure of ANA-12 required to block TrkB-receptors in the nucleus accumbens. In fact, the immobility time starts to decrease with the higher dose of ANA-12 in our experiments, albeit not reaching significance.

The selective serotonin reuptake inhibitor (SSRI) fluoxetine and the non-competitive antagonist of the N-methyl-D-aspartic acid (NMDA) glutamate receptor ketamine decreased the immobility time in agreement with previous studies (Detke and Lucki 1996; Maeng, et al. 2008; Koike, et al. 2011). The differences found between mouse and rat in the response to the effects of the selective serotonin reuptake blocker, fluoxetine could be due to species/strain-specific responses to the 5-HT transmission in the brain. In a study examining the effects of acute administration of fluoxetine on a physiological measure of anxiety, stress-induced hyperthermia, in rats and mice (Conley and Hutson 2007), acute fluoxetine elicited clear species-specific effects. Thus in mice, stress-induced hyperthermia and activity were unaffected by fluoxetine. In contrast, in rats, fluoxetine caused a significant baseline hypothermia in the absence of stress, confounding further interpretation. Moreover, slight differences in the pharmacokinetics and pharmacodynamics of fluoxetine in mice and rats could also be an influencing factor (Anelli et al. 1992).

In line with some earlier studies, the results of the present study also indicate that the effects of both ketamine and fluoxetine are, at least partly, mediated by BDNF/TrkB pathways since the antidepressant-like effect (i.e. decrease in immobility time in FST) of both ketamine and fluoxetine was significantly blocked by pre-treatment with the selective TrkB receptor antagonist ANA-12. Moreover, combined administration of a sub-threshold dose of the TrkB PAM AC-0026845 with ketamine or fluoxetine induced a significant additive or synergistic effect, suggesting a potential for mutual or linked pathways and possible co-treatment options.

Multiple lines of evidence show that brain-derived neurotrophic factor (BDNF), its specific receptor, TrkB, and subsequent mammalian target of rapamycin complex 1 (mTORC1) signaling play an important role in the pathophysiology of major depressive disorder (MDD), as well as in the therapeutic mechanisms of antidepressants (Nestler et al. 2002; Hashimoto et al. 2004; Duman and Monttegia 2006; Martinowich et al. 2007; Hashimoto 2010; Castren 2014; Lindholm and Castren 2014, Casarotto et al. 2021). In learned helplessness models of depression, a single infusion of BDNF into the dentate gyrus (DG) and CA3 pyramidal cell layers of the hippocampus results in long-lasting antidepressant effects (Shirayama et al. 2002). A viral-mediated gene transfer approach found that BDNF in the DG might be essential for mediating the therapeutic effect of antidepressants (Adachi et al. 2008). Another study showed that heterozygous BDNF^+/Met^ mice, carrying the human BDNF Val66Met polymorphism, exhibited decreased BDNF levels and apical dendritic spine density in the prefrontal cortex (PFC) after stress, which caused depression-like behavior (Yu et al. 2012). Furthermore, loss of BDNF in the forebrain attenuated the actions of an antidepressant (Monteggia et al. 2004), and responses elicited by antidepressants were lost in mice with either reduced brain BDNF levels or inhibited TrkB signaling (Saarelainen et al. 2003; Monteggia et al. 2007). These findings suggest that brain BDNF produces antidepressant-like effects in the hippocampus and PFC (Nestler et al. 2002; Shirayama et al. 2002; Duman and Monteggia 2006). Thus, the beneficial effects on depression-like behavior observed with Trk-PAM compounds in mice in the forced swim test may be related to activation of the BDNF/TrkB pathways, which has been suggested to play an important role in major depressive disorder.

Although several mechanisms may underlie the decrease of immobility time (considered as an index of antidepressant-like activity) following administration of the different Trk-PAM compounds in the FST, one plausible mechanism is the potentiation or enhancement of BDNF mediated signaling, a finding that was confirmed by our finding that pre-treatment of mice with the selective TrkB receptor antagonist ANA-12 completely blocked the antidepressant-like activity.

An additional mechanism behind the antidepressant-like effect of different positive allosteric modulators of BDNF/TrkB signaling in FST might be related to potential interaction of BDNF with the indoleamine, serotonin (5-HT) and/or monoamine, noradrenaline and dopamine systems. The monoamine theory of depression states that depression is associated with a decrease in monoamine levels in the synaptic cleft. The main neurotransmitter systems implicated in depression are the serotonin, noradrenaline and dopaminergic systems (Mongeau et al. 1997; Harro and Oreland 2001). However, an involvement of glutamatergic system in depression has also been strongly proposed (Duman and Aghajanian 2012; Mathew et al. 2012; Dolgin 2013; Browne and Lucki 2013). Our results indicate that there is a significant interaction between BDNF, serotonin and glutamatergic systems in FST. Acute administration of the non-competitive NMDA receptor antagonist, ketamine and the selective serotonin reuptake inhibitor, fluoxetine displayed a significantly shorter immobility time compared with control mice in FST, probably partly mediated by BDNF/TrkB signaling pathway, since the antidepressant-like effect of ketamine and fluoxetine was blocked by the selective TrkB receptor antagonist, ANA-12. Furthermore, combined administration of non-effective dose of Trk-PAM, AC-0026845 with ketamine or fluoxetine induced a significant additive or synergistic effect (a marked decrease in immobility time) compared with control group, fluoxetine- and AC-0026845-treated groups. Moreover, in vivo microdialysis data showed that ACD856 was able to significantly increase the amount of serotonin levels in the ventral hippocampus of freely moving rats, while noradrenaline and dopamine were less affected (Fig. 8).

The role of serotonin in depression is well known. Antidepressants, including the selective serotonin reuptake inhibitors (SSRIs) have been widely used as therapeutic drugs for MDD, although up to two thirds of patients fail to response to initial treatment. Interestingly, chronic administration of SSRIs have been reported to increase BDNF levels in the hippocampus and prefrontal cortex (PFC) (Hashimoto et al. 2004; Duman and Monteggia 2006; Martinowich et al. 2007; Hashimoto 2010; Duman and Aghajanian 2012), suggesting that BDNF/TrkB signaling is a part of the therapeutic antidepressant effects. Moreover, the TrkB receptor are expressed in the region of raphe nuclei (Madhav et al. 2001) where BDNF controls the survival and maintenance of developing serotonin neurons (Galter and Unsicker 2000). Previous studies have demonstrated that deletion of TrkB receptors from neurons in the median raphe region (serotonergic neurons) resulted in a loss of antidepressant efficacy and heightened aggression (Adachi et al. 2017).

Considering the emerging role of glutamate in the pathophysiology of MDD (Sanacora et al. 2008; Hashimoto 2009; Zarate et al. 2010; Krystal et al. 2013), it seems that the glutamatergic systems are integral to the emergence of depression-like behaviors in the FST. Interestingly, it was reported that the NMDA receptor antagonist, ketamine, showed a rapid antidepressant effect via increased BDNF levels (Autry et al. 2011), suggesting a role for BDNF-TrkB signaling in ketamine’s rapid antidepressant response. Recently, Casarotto, reported that Ketamine displays direct binding to TrkB and that allosteric facilitation of BDNF signaling is a common mechanism for antidepressant action, which may explain why typical antidepressant act slowly and how molecular effects of antidepressants are translated into clinical mood recovery (Casarotto et al. 2021). Therefore, it is likely that a potential interaction between BDNF signaling with glutamatergic and/or serotonergic systems are critical to the development of antidepressant-like behavior in FST, although further detailed studies are needed to confirm this.

We have previously reported on the positive effects of Trk-PAM compounds e.g. ACD856 on cognition. Cognitive dysfunction is also an integral part of the symptomatology in MDD. Currently, Vortioxetine, a compound with a broad effect on the serotonergic system, is the only approved treatment for MDD that has show positive cognitive effect in multiple domains (Perini et al. 2019). Therefore, Trk-PAM compounds such as ACD856 could provide a novel therapeutic avenue for MDD with potential for effects on multiple affected functions.

In conclusion, our study shows that several different compounds within the same structural series from Trk-PAM’s have antidepressant-like activity. It seems also that potential interaction between BDNF/TrkB signaling with glutamatergic and/or serotonergic systems may modulate the depression-related behavior. Hence, Trk-PAM’s may provide a new therapeutic modality for the treatment of Major depressive disorders.

## Data Availability

The authors confirm that the data supporting the findings of this study are available within the article.

## References

[CR1] Adachi M, Barrot M, Autry AE, Theobald D, Monteggia LM (2008). Selective loss of brain-derived neurotrophic factor in the dentate gyrus attenuates antidepressant efficacy. Biol Psychiatry.

[CR2] Adachi M, Autry AE, Mahgoub M, Suzuki K, Monteggia LM (2017). TrkB signaling in dorsal raphe nucleus is essential for antidepressant efficacy and normal aggression behavior. Neuropsychopharmacology.

[CR3] Ahlskog JE, Geda YE, Graff-Radford NR, Petersen RC (2011). Physical exercise as a preventive or disease-modifying treatment of dementia and brain aging. Mayo Clin Proc.

[CR4] Anelli M, Bizzi A, Caccia S, Codegoni AM, Fracasso C, Garattini S (1992). Anorectic activity of fluoxetine and norfluoxetine in mice, rats and guinea-pigs. J Pharm Pharmacol.

[CR5] Autry AE, Monteggia LM (2012). Brain-derived neurotrophic factor and neuropsychiatric disorders. Pharmacol Rev.

[CR6] Autry AE, Adachi M, Nosyreva E, Na ES, Los MF, Cheng P, Kavalali ET, Monteggia LM (2011). NMDA receptor blockade at rest triggers rapid behavioural antidepressant responses. Nature.

[CR7] Berman RM, Cappiello A, Anand A, Oren DA, Heninger GR, Charney DS (2000). Antidepressant effects of ketamine in depressed patients. Biol Psychiatry.

[CR8] Birkenhager TK, Geldermans S, Van den Broek WW, van Beveren N, Fekkes DJ (2012). Serum brain-derived neurotrophic factor level in relation to illness severity and episode duration in patients with major depression. Psychiatr Res.

[CR9] Borsini F, Meli A (1988). Is the forced swimming test a suitable model for revealing antidepressant activity?. Psychopharmacology.

[CR10] Browne CA, Lucki I (2013). Antidepressant effects of ketamine: mechanisms underlying fast-acting novel antidepressants. Front Pharmacol.

[CR11] Brunoni AR, Baeken C, Machado-Vieira R, Gattaz WF, Vanderhasselt MA (2014). BDNF blood levels after electroconvulsive therapy in patients with mood disorders: a systematic review and meta-analysis. World J Biol Psychiatry.

[CR12] Casarotto PC, Girych C, Fred SM, Kovaleva V, Moliner R, Enkavi G (2021). Antidepressant drugs act by directly binding to TRKB neurotrophin receptors. Cell.

[CR13] Castrén E (2014). Neurotrophins and psychiatric disorders. Handb Exp Pharmacol.

[CR14] Castrén E, Antila H (2017). Neuronal plasticity and neurotrophic factors in drug responses. Mol Psychiatry.

[CR15] Cazorla M, Prémont J, Mann A, Girard N, Kellendonk C, Rognan D (2011). Identification of a low-molecular weight TrkB antagonist with anxiolytic and antidepressant activity in mice. J Clin Invest.

[CR16] Conley RK, Hutson PH (2007). Effects of acute and chronic treatment with fluoxetine on stress-induced hyperthermia in telemetered rats and mice. Eur J Pharmacol.

[CR17] Cowansage KK, LeDoux JE, Monfils M-H (2010). Brain-derived neurotrophic factor: a dynamic gatekeeper of neural plasticity. Curr Mol Pharmacol.

[CR18] Cryan JF, Holmes A (2005). The ascent of mouse: advances in modelling human depression and anxiety. Nat Rev Drug Discovery.

[CR19] Cryan JF, Page ME, Lucki I (2005). Differential behavioral effects of the antidepressants reboxetine, fluoxetine, and moclobemide in a modified forced swim test following chronic treatment. Psychopharmacology.

[CR20] Cryan JF, Valentino RJ, Lucki I (2005). Assessing substrates underlying the behavioral effects of antidepressants using the modified rat forced swimming test. Neurosci Biobehav Rev.

[CR21] Czéh B, Fuchs E, Wiborg O, Simon M (2016). Animal models of major depression and their clinical implications. Prog Neuropsychopharmacol Biol Psychiatry.

[CR22] Dahlström M, Madjid N, Nordvall G, Halldin M, Vazquez-Juarez E, Lindskog M, Sandin J, Winblad B, Eriksdotter M, Forsell P (2021). Identification of Novel Positive Allosteric Modulators of Neurotrophin Receptors for the Treatment of Cognitive Dysfunction. Cells.

[CR23] Dalvi A, Lucki I (1999). Murine models of depression. Psychopharmacology.

[CR24] Detke MJ, Lucki I (1996). Detection of serotonergic and noradrenergic antidepressants in the rat forced swimming test: the effects of water depth. Behav Brain Res.

[CR25] Diaz-Granados N, Ibrahim LA, Brutsche NE, Ameli R, Henter ID, Luckenbaugh DA, Machado-Vieira R, Zarate CA (2010). Rapid resolution of suicidal ideation after a single infusion of an N-methyl-D-aspartate antagonist in patients with treatment-resistant major depressive disorder. J Clin Psychiatry.

[CR26] Dolgin E (2013). Rapid Antidepressant Effects of Ketamine Ignite Drug Discovery.

[CR27] Dording CM, Mischoulon D, Petersen TJ, Kornbluh R, Gordon J, Nierenberg AA (2002). The pharmacologic management of SSRI-induced side effects: a survey of psychiatrists. Ann Clin Psychiatry.

[CR28] Duman RS, Aghajanian GK (2012). Synaptic dysfunction in depression: potential therapeutic targets. Science.

[CR29] Duman RS, Monteggia LM (2006). A neurotrophic model for stress-related mood disorders. Biol Psychiatry.

[CR30] Egan MF, Kojima M, Callicott JH, Goldberg TE, Kolachana BS, Bertolino A (2003). The BDNF val66met polymorphism affects activity-dependent secretion of BDNF and human memory and hippocampal function. Cell.

[CR31] Engesser-Cesar C, Anderson AJ, Cotman CW (2007). Wheel running and fluoxetine antidepressant treatment have differential effects in the hippocampus and the spinal cord. Neuroscience.

[CR32] Fava M, Rush AJ, Alpert JE, Balasubramani GK, Wisniewski SR, Carmin CN (2008). Difference in treatment outcome in outpatients with anxious versus nonanxious depression: a STAR∗D report. Am J Psychiatry.

[CR33] Frazer A, David A, Morilak DA (2005). What should animal models of depression model?. Neurosci Biobehav Rev.

[CR34] Galter D, Unsicker K (2000). Brain-derived neurotrophic factor and trkB are essential for cAMP-mediated induction of the serotonergic neuronal phenotype. J Neurosci Res.

[CR35] Gelenberg AJ, Chesen CL (2000). How fast are antidepressants?. J Clin Psychiatry.

[CR36] Harro J, Oreland L (2001). Depression as a spreading adjustment disorder of monoaminergic neurons: a case for primary implication of the locus coeruleus. Brain Res Rev.

[CR37] Hashimoto K (2009). Emerging role of glutamate in the pathophysiology of major depressive disorder. Brain Res Rev.

[CR38] Hashimoto K (2010). Brain-derived neurotrophic factor as a bio- marker for mood disorders: an historical overview and future directions. Psychiatry Clin Neurosci.

[CR39] Hashimoto K, Shimizu E, Iyo M (2004). Critical role of brain- derived neurotrophic factor in mood disorders. Brain Res Rev.

[CR40] Kehr J, Dechent P, Kato T, Ogren SO (1998). Simultaneous determination of acetylcholine, choline and physostigmine in microdialysis samples from rat hippocampus by microbore liquid chromatography/electrochemistry on peroxidase redox polymer coated electrodes. J Neurosci Methods.

[CR41] Kehr J, Hu X, Yoshitake T, Scheller D (2007). Determination of the dopamine agonist rotigotine in microdialysates from the rat brain by microbore column liquid chromatography with electrochemical detection. J Chromatogr.

[CR42] Kessler RC, Berglund P, Demler O, Jin R, Koretz D, Merikangas KR (2003). The epidemiology of major depressive disorder: results from the National Comorbidity Survey Replication (NCS-R). JAMA.

[CR43] Kessler RC, Chiu WT, Demler O, Merikangas KR, Walters EE (2005). Prevalence, severity, and comorbidity of 12-month DSM-IV disorders in the National Comorbidity Survey Replication. Arch Gen Psychiatry.

[CR44] Khakpai F, Ebrahimi-Ghiri M, Alijanpour S, Zarrindast MR (2019). Ketamine-induced antidepressant like effects in mice: A possible involvement of cannabinoid system. Biomed and Pharmacotherapy.

[CR45] Kim YK, Lee HP, Won SD, Park EY, Lee HY, Lee BH (2007). Low plasma BDNF is associated with suicidal behavior in major depression. Prog Neuropsychopharmacol Biol Psychiatry.

[CR46] Koike H, Iijima M, Chaki S (2011). Involvement of AMPA receptor in both the rapid and sustained antidepressant-like effects of ketamine in animal models of depression. Behav Brain Res.

[CR47] Krystal JH, Sanacora G, Dunman RS (2013). Rapid-acting glutamatergic antidepressants: The path to ketramine and beyond. Biol Psychiatry.

[CR48] Kuteeva E, Hökfelt T, Ogren SO (2005). Behavioural characterisation of young adult transgenic mice overexpressing galanin under the PDGF-B promoter. Regul Pept.

[CR49] Lam RW, Kennedy SH (2004). Evidence-based strategies for achieving and sustaining full remission in depression: focus on metaanalyses. Canadian journal of psychiatry. Revue Canadienne De Psychiatrie.

[CR50] Lee BH, Kim K (2010). The roles of BDNF in the pathophysiology of major depression and in antidepressant treatment. Psychiatry Investig.

[CR51] Lee BH, Kim H, Park SH, Kim YK (2007). Decreased plasma BDNF level in depressive patients. J Affect Disord.

[CR52] Lepack AE, Fuchikami M, Dwyer JM, Banasr M, Duma RS (2014) BDNF Release Is Required for the Behavioral Actions of Ketamine. Int J Neuropsychopharmacol 18(1):1–6 10.1093/ijnp/pyu033 PMC4368871 25539510

[CR53] Licinio J, Wong ML (2005). Depression, antidepressants and suicidality: a critical appraisal. Nat Rev Drug Discov.

[CR54] Lindholm JS, Castrén E (2014). Mice with altered BDNF signaling as models for mood disorders and antidepressant effects. Front Behav Neurosci.

[CR55] Liu RJ, Lee FS, Li XY, Bambico F, Duman RS, Aghajanian GK (2012). Brain-derived neurotrophic factor Val66Met allele impairs basal and ketamine-stimulated synaptogenesis in prefrontal cortex. Biol Psychiatry.

[CR56] Lucki I, Dalvi A, Mayorga AJ (2001). Sensitivity to the effects of pharmacologically selective antidepressants in different strains of mice. Psychopharmacology.

[CR57] Madhav TR, Pei Q, Zetterström TS (2001). Serotonergic cells of the rat raphe nuclei express mRNA of tyrosine kinase B (trkB), the high-affinity receptor for brain derived neurotrophic factor (BDNF). Brain Res Mol Brain Res.

[CR58] Maeng S, Zarate CA, Du J, Schloesser RJ, McCammon J, Chen G, Manji HK (2008). Cellular mechanisms underlying the antidepressant effects of ketamine: role of alpha-amino-3-hydroxy-5-methylisoxazole-4-propionic acid receptors. Biol Psychiatry.

[CR59] Mann JJ (1999). Role of the serotonergic system in the pathogenesis of major depression and suicidal behavior. Neuropsychopharmacology.

[CR60] Martinowich K, Manji H, Lu B (2007). New insights into BDNF function in depression and anxiety. Nat Neurosci.

[CR61] Mathew SJ, Shah A, Lapidus K, Clark C, Jarun N, Ostermeyer B (2012). Ketamine for treatment-resistant unipolar depression: current evidence. CNS Drugs.

[CR62] Matrisciano F, Bonaccorso S, Ricciardi A, Scaccianoce S, Panaccione I, Wang L (2009). Changes in BDNF serum levels in patients with major depression disorder (MDD) after 6 months treatment with sertraline, escitalopram, or venlafaxine. J Psychiatr Res.

[CR63] Mikael T, Katarina V, Yoshiro O, Lundberg J (2018). The 5-HT1B receptor – a potential target for antidepressant treatment. Psychopharmacology.

[CR64] Molendijk ML, Spinhoven P, Polak M, Bus BA, Penninx BW, Elzinga BM (2014). Serum BDNF concentrations as peripheral manifestations of depression: evidence from a systematic review and meta-analyses on 179 associations (N=9484). Mol Psychiatry.

[CR65] Mongeau R, Blier P, de Montigny C (1997). The serotonergic and noradrenergic systems of the hippocampus: their interactions and the effects of antidepressant treatments. Brain Res Rev.

[CR66] Monteggia LM, Barrot M, Powell CM, Berton O, Galanis V, Gemelli T (2004). Essential role of brain-derived neurotrophic factor in adult hippocampal function. Proc Natl Acad Sci USA.

[CR67] Monteggia LM, Luikart B, Barrot M, Theobold D, Malkovska I, Nef S, Parada LF, Nestler EJ (2007). Brain-derived neurotrophic factor conditional knockouts show gender differences in depression-related behaviors. Biol Psychiatry.

[CR68] Nestler EJ, Barrot M, DiLeone RJ, Eisch AJ, Gold SJ, Monteggia LM (2002). Neurobiology of depression. Neuron.

[CR69] Overstreet DH, Friedman E, Mathe AA, Yadid G (2005). The Flinders Sensitive Line rat: a selectively bred putative animal model of depression. Neurosci Biobehav Rev.

[CR70] Perini G, Cotta Ramusino M, Sinforiani E, Bernini S, Petrachi R, Costa A (2019). Cognitive impairment in depression: recent advances and novel treatments. Neuropsychiatr Dis Treat.

[CR71] Petryshen TL, Lewis MC, DennehyKA GJC, Fava M (2016). Antidepressant-like effect of low dose ketamine and scopolamine co-treatment in mice. Neurosci Lett.

[CR72] Porsolt RD, Le Pichon M, Jalfre M (1977). Depression: a new animal model sensitive to antidepressant treatments. Nature.

[CR73] Porsolt RD, Anton G, Blavet N, Jalfre M (1978). Behavioural despair in rats: a new model sensitive to antidepressant treatments. Eur J Pharmacol.

[CR74] Qi Z, Fieni D, Tretter F, Voit EO (2013). The neurochemical mobile with non-linear interaction matrix: an exploratory computational model. Pharmacopsychiatry.

[CR75] Qi Z, Gina PY, Tretter F, Pogarell O, Grace AA, Voit EO (2016). A heuristic model for working memory deficit in schizophrenia. Biochim Biophys Acta Gen Subj.

[CR76] Reardon S (2019). Depression researchers rethink popular mouse swim tests. Nature.

[CR77] Saarelainen T, Hendolin P, Lucas G, Koponen E, Sairanen M, MacDonald E (2003). Activation of the TrkB neurotrophin receptor is induced by antidepressant drugs and is required for antidepressant-induced behavioral effects. J Neurosci.

[CR78] Sanacora G, Zarate CA, Krystal JH, Manji KH (2008) Targeting the glutamatergic system to develop novel, improved therapeutics for mood disorders. Nat Rev Drug Discov 7(5):426–437 10.1038/nrd2462 PMC2715836 18425072

[CR79] Sánchez MG, Bourque M, Morissette M, Di Paolo T (2010). Steroids-Dopamine Interactions in the Pathophysiology and Treatment of CNS Disorders. CNS Neurosci Ther.

[CR80] Seligman ME, Weiss J, Weinraub M, Schulman A (1980). Coping behavior: learned helplessness, physiological change and learned inactivity. Behav Res Ther.

[CR81] Sghendo L, Mifsud J (2012). Understanding the molecular pharmacology of the serotonergic system: using fluoxetine as a model. J Pharm Pharmacol.

[CR82] Shirayama Y, Chen AC-H, Nakagawa S, Russell DS, Duman RS (2002). Brain-derived neurotrophic factor produces antide- pressant effects in behavioral models of depression. J Neurosci.

[CR83] Steru L, Chermat R, Thierry B, Simon P (1985). The tail suspension test: a new method for screening antidepressants in mice. Psychopharmacology.

[CR84] Thase ME (2006). Managing depressive and anxiety disorders with escitalopram. Expert Opin Pharmacother.

[CR85] Trivedi MH, Fava M, Wisniewski SR, Thase ME, Quitkin F, Warden D (2006). Medication augmentation after the failure of SSRIs for depression. N Engl J Med.

[CR86] Willner P (1997). Validity, reliability and utility of the chronic mild stress model of depression: a 10-year review and evaluation. Psychopharmacology.

[CR87] Yoshida T, Ishikawa M, Niitsu T, Nakazato M, Watanabe H, Shiraishi T (2012). Decreased serum levels of mature brain-derived neurotrophic factor (BDNF), but not its precursor proBDNF, in patients with major depressive disorder. PLoS ONE.

[CR88] Yoshitake T, Yoshitake S, Fujino K, Nohta H, Yamaguchi M, Kehr J (2004). High-sensitive liquid chromatographic method for determination of neuronal release of serotonin, noradrenaline and dopamine monitored by microdialysis in the rat prefrontal cortex. J Neurosci Methods.

[CR89] Yu H, Wang DD, Wang Y, Liu T, Lee FS, Chen ZY (2012). Variant brain-derived neurotrophic factor Val66Met polymorphismalters vulnerability to stress and response to antidepressants. J Neurosci.

[CR90] Zarate CA, Singh JB, Carlson PJ, Brutsche NE, Ameli R, Luckenbaugh DA, Charney DS, Manji HK (2006). A randomized trial of an N-methyl-D-aspartate antagonist in treatment-resistant major depression. Arch Gen Psychiatry.

[CR91] Zarate CA, Machado-Vieira R, Henter I, Ibrahim L, Diazgranados N, Salvadore G (2010). Glutamatergic modulators: The future of treating mood disorders?. Harv Rev Psychiatry.

[CR92] Zhang JC, Wu J, Fujita Y, Yao W, Ren Q, Yang C, Li SX, Shirayama Y, Hashimoto K (2014). Antidepressant effects of TrkB ligands on depression-like behavior and dendritic changes in mice after inflammation. Int J Neuropsychopharmacol..

